# Advanced atomic force microscopy techniques V

**DOI:** 10.3762/bjnano.16.6

**Published:** 2025-01-21

**Authors:** Philipp Rahe, Ilko Bald, Nadine Hauptmann, Regina Hoffmann-Vogel, Harry Mönig, Michael Reichling

**Affiliations:** 1 Institut für Physik, Universität Osnabrück, Barbarastraße 7, 49076 Osnabrück, Germany; 2 Institute of Chemistry, Hybrid Nanostructures, University of Potsdam, Karl-Liebknecht-Straße 24–25, 14476 Potsdam, Germany; 3 Institute for Molecules and Materials, Radboud University, 6525 AJ Nijmegen, The Netherlands; 4 Institute of Physics and Astronomy, University of Potsdam, Karl-Liebknecht-Str. 24–25, 14476 Potsdam, Germany; 5 Physikalisches Institut, Universität Münster, Wilhelm-Klemm-Str. 10, 48149 Münster, Germany

**Keywords:** AFM, atomic force microscopy, conductivity, drift correction, force spectroscopy, NC-AFM, non-contact atomic force microscopy, resistivity, tip–surface interaction

With the restrictions on travelling and social distancing lifted, we were delighted to continue two series of meetings on atomic force microscopy (AFM), the 23rd International Conference on Non-Contact Atomic Force Microscopy (NC-AFM) held in Nijmegen (Netherlands) and the 6th International Workshop on Advanced Atomic Force Microscopy Techniques held in Potsdam (Germany). The strong advance in the field and the high quality of the presentations motivated us to establish this thematic issue in the *Beilstein Journal of Nanotechnology* for compiling the latest results on developments and applications of atomic force microscopy techniques.

Atomic force microscopy, a technique soon celebrating its 40th anniversary, is nowadays a well-established tool for the investigation of nanoscale phenomena. The technique is steadily developed to increase accuracy, precision, and versatility; some of these developments are presented in contributions to this thematic issue.

Along these lines, Dickbreder et al. address the precision of scanning probe microscopy experiments by introducing a software for lateral drift correction [1]. Post-data acquisition drift correction for longer series of consecutively recorded image sets can be cumbersome and extremely time consuming. Here, the authors develop an easy-to-use and robust software tool (”unDrift”), which allows reliable and fast drift correction. Dickbreder et al. demonstrate the robust performance of the software tool by AFM data recorded under varying conditions (vacuum or liquid environment) on calcite surfaces with recording times up to several hours.

The work by Nony et al. addresses the significantly increased precision of force spectroscopy measurements when performed with a quartz cantilever allowing to reduce the oscillation amplitude to values in the low picometer regime [2]. As the conversion of frequency-shift to force data critically depends on the accurate knowledge of the quartz cantilever stiffness, the authors develop a method to quantify the stiffness based on thermal noise measurements and numerical simulation.

Calibrated measurements of conductivity and resistivity are the focus of the contribution by Piquemal et al. [3]. A particular challenge in precision measurements of local electrical parameters lies on possible contributions of environmental factors as well as on unknown electrical properties of the scanning tip. The work by Piquemal et al. tackles these challenges by introducing a reference sample suitable for calibrating the microscope over a wide current range. A central zone of the sample offers several contact pads, each addressable by the tip of a conductive atomic force microscopy probe, and each connected to a different calibrated resistor. Thus, calibrated current measurements over a large current range become possible.

Khachartryan et al. highlight the strength of cantilever displacement detection with a Michelson-type fibre interferometer and provide a model for interferometric signal generation [4]. The interferometer response is slightly nonlinear under typical NC-AFM working conditions, while a large cantilever oscillation amplitude yields a signal with a complex temporal structure. This is due to the interferometer signal being limited in amplitude by the spatial periodicity of the cavity light field. By the fit of a model function to the measured time-domain interferometer signal, all displacement signal parameters can precisely be determined regardless of the oscillation amplitude. As the periodicity of the cavity light field scales with the length standard given by the laser light wavelength, this specifically facilitates a calibration of the oscillation amplitude with unprecedented accuracy and precision not involving any tip–surface interaction.

The measurement of electrostatic properties at the nanoscale emerged as a most relevant subfield of atomic force microscopy, especially driven by electrostatic force microscopy (EFM), Kelvin probe force microscopy (KPFM), and closely related techniques.

Grévin et al. further push the boundaries of the detection by implementing an open-loop variant of KPFM which accesses the spectrum of a time-periodic surface potential [5]. By exploiting a double heterodyne frequency mixing effect, they can selectively transfer each harmonic component to the second cantilever eigenmode, which is particularly relevant when generating the time-periodic potential by optical or electrical pumping. With this development, the authors could present the detection of modulated components that are below the detection limit of other KPFM measurement modes.

Da Lisca et al. investigate the cross-sectional potential distribution across a III-V multilayer stack [6]. With a spatial resolution down to 20 nm at ambient conditions, they identified the presence of several space charge regions along the stack. The authors further conclude on future requirements on electrical contacts to carry out a more detailed characterization of the optoelectronic properties.

Rothhardt et al. map the local work function on graphene nanoribbons [7]. They experimentally investigate the charge transfer between a gold substrate and graphene nanoribbons and compare that to DFT calculations. Indeed, the doping of the graphene nanoribbons is reflected by the local work function. They also measure and calculate the local work function as a function of tip–sample distance and compare results to those of simple electrostatic models of a graphene nanoribbon, validating the overall approach of measurement and calculations.

Eftekari et al. measure the local surface photovoltage generated in a silicon photodiode integrated with a piezoelectric membrane [8]. The design of such a device allows for the laterally resolved simultaneous quantification of the photovoltage generated by the photodiode as well as the mechanical oscillation of the piezoelectric membrane with highest resolution in real time.

In addition to the measurement of surface potentials or photovoltages, Navarro-Rodriguez et al. investigate the dynamics of surface charges and how they couple to the detection system [9]. They describe in detail how Joule dissipation leads to energy dissipation of the cantilever oscillation and a reduction in amplitude for constant excitation. They focus on two-dimensional materials and discuss how the reduction in amplitude resulting from energy dissipation influences the height measurement. In addition to the inaccuracy caused by electrostatic or capillary forces, this is an additional mechanism having an impact on AFM height measurements.

Closely related is the measurement of conductivity. Skolaut et al. investigate conductivity in dependence on the roughness of the substrate using alkanethiol self-assembled monolayers (SAMs) and conducting AFM [10]. The authors find that rougher surfaces lead to stronger variations in conductivity, and it is suggested that a correlation of topography and conductivity maps is carried out to identify suitable areas for a representative averaging of conductivity values.

Müller et al. present the application of AFM-based infrared nanospectroscopy to coated polymer surfaces [11]. The authors prepare thin films of SiO*_x_* on polypropylene surfaces by plasma-enhanced chemical vapor deposition (PE-CVD), which is commonly done to improve gas barrier properties of polypropylene. They characterize SiO*_x_* films as thin as 5 nm by the so-called surface-sensitive mode, in contrast to the established contact mode, which provides much less surface sensitivity.

A particular strength for the study of materials is the combination of different measurement modes. Rothe et al. extend the characterization of defects in a single layer of graphene on iridium that were induced by rare-gas ion bombardment by using combined scanning tunnelling microscopy (STM) measurements and NC-AFM [12]. The authors reveal that presumed monoatomic vacancies, as deduced from STM measurements alone, have rather different origins. The authors assign one type of defects to a possible defect in the Ir surface. The other type is identified as four missing carbon atoms corroborated by a higher reactivity with the tip.

We thank all authors who contributed to this thematic issue and we are grateful to all reviewers for their input which was most helpful. It has been our greatest pleasure to work with the team at the *Beilstein Journal of Nanotechnology* and we appreciate their continuous support.

Philipp Rahe, Ilko Bald, Nadine Hauptmann, Regina Hoffmann-Vogel, Harry Mönig and Michael Reichling

Osnabrück, Potsdam, Nijmegen, and Münster, December 2024

## Data Availability

Data sharing is not applicable as no new data was generated or analyzed in this study.

## References

[1] Dickbreder T, Sabath F, Höltkemeier L, Bechstein R, Kühnle A (2023). Beilstein J Nanotechnol.

[2] Nony L, Clair S, Uehli D, Herrero A, Themlin J-M, Campos A, Para F, Pioda A, Loppacher C (2024). Beilstein J Nanotechnol.

[3] Piquemal F, Kaja K, Chrétien P, Morán-Meza J, Houzé F, Ulysse C, Harouri A (2023). Beilstein J Nanotechnol.

[4] Khachatryan K, Anter S, Reichling M, von Schmidsfeld A (2024). Beilstein J Nanotechnol.

[5] Grévin B, Husainy F, Aldakov D, Aumaître C (2023). Beilstein J Nanotechnol.

[6] da Lisca M, Alvarez J, Connolly J P, Vaissiere N, Mekhazni K, Decobert J, Kleider J-P (2023). Beilstein J Nanotechnol.

[7] Rothhardt D, Kimouche A, Klamroth T, Hoffmann-Vogel R (2024). Beilstein J Nanotechnol.

[8] Eftekhari Z, Rezaei N, Stokkel H, Zheng J-Y, Cerreta A, Hermes I, Nguyen M, Rijnders G, Saive R (2023). Beilstein J Nanotechnol.

[9] Navarro-Rodriguez M, Somoza A M, Palacios-Lidon E (2024). Beilstein J Nanotechnol.

[10] Skolaut J, Tepper J, Galli F, Wulfhekel W, van Ruitenbeek J M (2023). Beilstein J Nanotechnol.

[11] Müller H, Stadler H, de los Arcos T, Keller A, Grundmeier G (2024). Beilstein J Nanotechnol.

[12] Rothe K, Néel N, Kröger J (2024). Beilstein J Nanotechnol.

